# An ionizable lipid toolbox for RNA delivery

**DOI:** 10.1038/s41467-021-27493-0

**Published:** 2021-12-13

**Authors:** Xuexiang Han, Hanwen Zhang, Kamila Butowska, Kelsey L. Swingle, Mohamad-Gabriel Alameh, Drew Weissman, Michael J. Mitchell

**Affiliations:** 1 Department of Bioengineering, University of Pennsylvania, Philadelphia, PA 19104 USA; 2 Laboratory of Biophysics, Intercollegiate Faculty of Biotechnology, University of Gdańsk and Medical University of Gdańsk, Abrahama 58, 80-307 Gdańsk, Poland; 3 Department of Medicine, University of Pennsylvania, Philadelphia, PA 19104 USA; 4 Abramson Cancer Center, Perelman School of Medicine, University of Pennsylvania, Philadelphia, PA 19104 USA; 5 Institute for Immunology, Perelman School of Medicine, University of Pennsylvania, Philadelphia, PA 19104 USA; 6 Cardiovascular Institute, Perelman School of Medicine, University of Pennsylvania, Philadelphia, PA 19104 USA; 7 Institute for Regenerative Medicine, Perelman School of Medicine, University of Pennsylvania, Philadelphia, PA 19104 USA

**Keywords:** Nucleic-acid therapeutics, Drug delivery, DNA and RNA

## Abstract

Recent years have witnessed incredible growth in RNA therapeutics, which has benefited significantly from decades of research on lipid nanoparticles, specifically its key component—the ionizable lipid. This comment discusses the major ionizable lipid types, and provides perspectives for future development.

## Abstract (web summary)

RNA therapeutics have benefited significantly from decades of research on lipid nanoparticles, specifically its key component—the ionizable lipid. This comment discusses the major ionizable lipid types, and provides perspectives for future development.

## Need for ionizable lipids

**Fig. 1:**
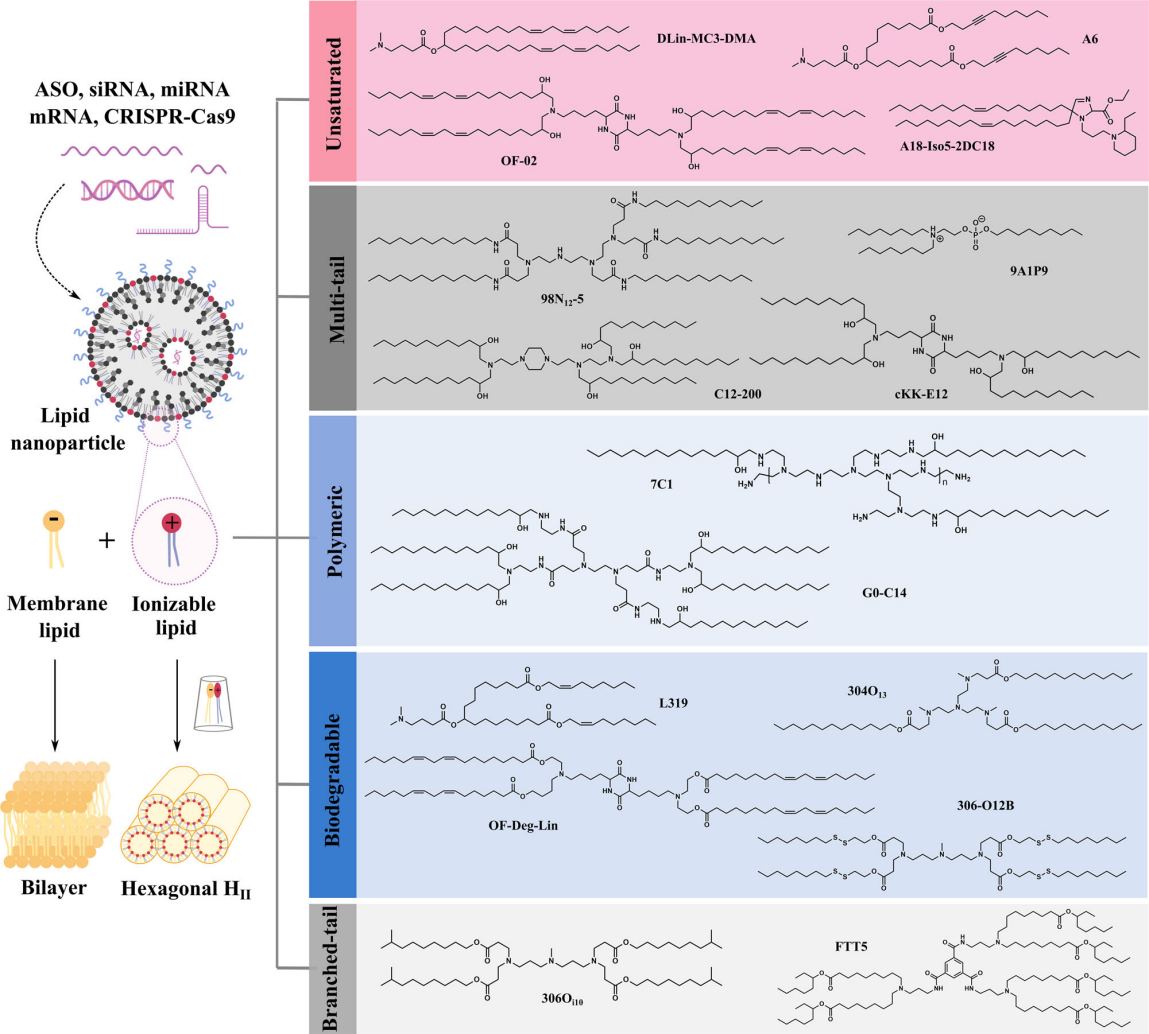
Mechanism for endosome disruption by ionizable lipids and five major structural classes of ionizable lipids. The cone-shaped ion pairs formed by anionic endosomal phospholipids and protonated ionizable lipids can disrupt the bilayer structure to promote endosomal escape. Based on their structural properties, RNA-delivering ionizable lipids can be categorized into unsaturated (containing unsaturated bond), multi-tail (containing more than two tails), polymeric (containing polymer or dendrimer), biodegradable (containing biodegradable bond) and branched-tail (containing branched tail) ones.

Broadly speaking, ribonucleic acid (RNA) therapeutics include antisense oligonucleotides (ASOs), small interfering RNAs (siRNAs), microRNAs (miRNAs), messenger RNAs (mRNAs), and single-guide RNAs (sgRNAs)-mediated CRISPR-Cas9 system, which can manipulate essentially any gene of interest through distinct modes of action^1^. However, RNA therapeutics are susceptible to nucleases and cannot permeate cells due to their large size and negative charge. Delivery of RNAs to target cells by clinically translatable lipid nanoparticles (LNPs) provides vast opportunities to tackle a series of life-threatening diseases including COVID-19^2^. LNPs typically consist of four components—ionizable lipid, phospholipid, cholesterol, and PEGylated lipid, among which, the ionizable lipid plays a major role in protecting RNAs and facilitating their cytosolic transport. Ionizable lipids are positively charged at acidic pH to condense RNAs into LNPs, but are neutral at physiological pH to minimize toxicity. They can be protonated in the acidic endosome after cellular uptake, and interact with anionic endosomal phospholipids to form cone-shaped ion pairs that are not compatible with a bilayer (Fig. 1). These cationic-anionic lipid pairs drive the transition from the bilayer structure to the inverted hexagonal H_II_ phase, which facilitates membrane fusion/disruption, endosomal escape and cargo release into the cytosol^3^. Since 2008, ionizable lipids with diverse chemical identities have been created. Systematic categorization of these lipids based on their structures can greatly benefit the field and facilitate the development of next-generation ionizable lipids. Currently, there are five major ionizable lipid types that are widely used for RNA delivery (Fig. 1).

## Unsaturated ionizable lipids

Tail saturation greatly influences the fluidity and delivery efficiency of ionizable lipids. Increasing the tail unsaturation from 0 to 2 *cis* double bonds correlates with an increased tendency for bilayer lipids to form a nonbilayer phase^4^, leading to enhanced membrane disruption and payload release. For this reason, the linoleyl tail was chosen as the structural basis during the optimization process of the ionizable lipid **DLin-MC3-DMA** (**MC3**)^3,5^. This ionizable lipid confers robust hepatic gene silencing and is used in the first FDA-approved siRNA drug Onpattro^®^ for the treatment of hereditary transthyretin amyloidosis (hATTR)^6^. Importantly, both the structure and formulation of **MC3** laid the groundwork for further LNP development.

Unsaturated ionizable lipids have also been demonstrated to enhance mRNA delivery. Anderson and colleagues showed a linoleic acid-derived ionizable lipid (**OF-02**) achieved the highest hepatic mRNA delivery and protein expression among its class^7^. Later, they demonstrated that an alkyne ionizable lipid (**A6**) could greatly increase membrane fusion and facilitate albumin-mediated mRNA delivery when incorporated into benchmark LNPs (e.g., **MC3**)^8^. Interestingly, in a heterocyclic ionizable lipid library prepared by a three-component reaction, they identified an alkylene ketone-derived **A18-Iso5-2DC18** that could not only deliver mRNA vaccines robustly but also activate the stimulator of interferon genes (STING) pathway^9^. However, the inclusion of unsaturated bonds in the ionizable lipid does not always correspond with potent in vivo RNA delivery^7,8,10^, indicating that both rational design and screening are necessary.

## Multi-tail ionizable lipids

Multi-tail ionizable lipids are structurally distinguished from two-tail ones by having three or more tails. Such ionizable lipids are expected to produce a more cone-shaped structure with enhanced endosome-disrupting ability due to the increased cross-section of the tail region. Multi-tail ionizable lipids can be easily synthesized by combinatorial chemistry and subjected to high-throughput screening. Since the first combinatorial library created in 2008, several multi-tailed leads (e.g., **98N**_**12**_**-5**, **C12-200**, and **cKK-E12**) have been identified to potently knockdown hepatic genes^11–13^.

Although initially developed for siRNA delivery, multi-tail ionizable lipids can be repurposed for efficient mRNA delivery by optimizing the LNP composition via design of experiment methodologies with significantly reduced workload^14,15^. For example, the optimized formulation of **C12-200** increased mRNA expression 7-fold compared to the standard formulation^14^. Later, this formulation was widely adopted by multi-tail ionizable lipids for various mRNA delivery purposes such as chimeric antigen receptor T-cell engineering and prenatal protein replacement therapy^16,17^. Recently, a class of multi-tail ionizable phospholipids was created; the top-performing **9A1P9**, comprising one zwitterionic head and three tails, assisted to promote membrane destabilization and cargo release, which greatly enhanced LNP-mediated tissue-selective mRNA delivery and gene editing^18^. Nevertheless, lead multi-tail ionizable lipids often have stable backbones and low degradability, so their toxicity and immunogenicity should always be taken into consideration^19^.

## Ionizable polymer-lipids

Substitution of free amines on cationic polymers with alkyl tails affords ionizable polymer-lipids, which can enhance particle formation through hydrophobic aggregation. From 500 structurally diverse synthetic polymer-lipid hybrids, Anderson et al. identified that C15 epoxide-modified low-molecular-weight polyethyleneimine (**7C1**) conferred the most efficient non-liver siRNA delivery^20^. **7C1** preferentially transfected endothelial cells—rather than hepatocytes and pulmonary epithelial cells—in multiple organs, with the highest endothelial gene silencing in the lung. In non-human primates, ~80% endothelial gene knockdown in the lungs was achieved by **7C1** without significant toxicity^21^, indicating its potential to treat dysfunctional endothelium-related diseases. Recently, its re-optimized formulation was further demonstrated to achieve potent gene silencing in bone marrow endothelial cells, which could further regulate hematopoietic cell activity^22^.

Additionally, lead ionizable polymer-lipids derived from poly(amido amine) and poly(propylenimine) dendrimers have also shown strong avidity to transfect liver endothelium and lung vasculature^23,24^, implying preferential transfection of endothelium for polycation-based ionizable lipids. However, such cell population tropism can be varied depending on the LNP formulation^23,25^. For example, when co-formulated with other accessory excipients, **G0-C14** confers high accumulation and effective transfection of various RNA therapeutics in tumors^26–29^, demonstrating the promise of LNPs for cancer therapy. Nevertheless, ionizable polymer-lipids, even after purification, usually comprise a mixture of different substitution compounds^20,23^, which increase their complexity. Moreover, the toxic polycation core and non-degradable backbone pose extra hurdles for clinical translation.

## Biodegradable ionizable lipids

In order to reduce accumulation and potential side effects, ionizable lipids should be readily degraded into non-toxic metabolites after successful intracellular cargo delivery, which is especially crucial for RNA therapeutics requiring repeated dosing. A common strategy to introduce biodegradability into ionizable lipids is through the inclusion of ester bonds that are stable at physiological pH, but are enzymatically hydrolyzed within tissues and cells. For example, due to the slow degradability of the dilinoleyl tail in **MC3**, a biodegradable substitution (**L319**) was created by replacing one of the double bonds in each tail with a primary ester^30^. **L319** not only maintained in vivo potency, but also displayed rapid elimination and improved tolerability. Notably, the position and steric effect of the ester groups can greatly affect ionizable lipid clearance and potency^30,31^.

Alternative to rational design, diversely structured biodegradable ionizable lipids can be combinatorially synthesized using alkyl amines and acrylate tails^11,32^. The analysis of structure–activity relationships indicated that acrylate-based ionizable lipids with tertiary amines, at least three O_13_ tails, and a suitable p*K*_a_ (5.5–7.0) confer robust in vivo gene silencing^32^. The lead ionizable lipid (**304O**_**13**_) had similar potency to the non-degradable **C12-200** benchmark, but posed much lower toxicity at high doses. It is noteworthy that ester-containing ionizable lipids typically demonstrate lower potency compared to non-degradable analogs due to inefficient delivery caused by rapid hydrolysis^3,11^. This suggests an activity-degradability tradeoff that needs to be balanced to maximize total benefits. One viable approach to overcome this dilemma is adopting the more stable secondary ester instead of the primary one^31^. Interestingly, **OF-Deg-Lin**, a degradable analog of **OF-02**, selectively transfected splenic lymphocytes and induced >85% of total protein expression in the spleen (an organ with a relative low-abundance of hydrolases)^33^, suggesting that degradable ionizable lipids could be promising vectors for spleen transfection.

To further improve biodegradability and accelerate RNA release, disulfide bonds that are sensitive to the reductive intracellular environment can be incorporated into acrylate tails^34^. The resulting bioreducible ionizable lipids have been shown to efficiently deliver ASOs and CRISPR/Cas9 systems for gene silencing and editing with good tolerability in mice^35–37^. For example, **306-O12B** outperformed **MC3** in CRISPR-Cas9–based liver genome editing of *Angptl3*, and negligible toxicity or off-target mutagenesis was observed^38^. Despite the promise of disulfide bond-containing ionizable lipids, the difficulty of synthesis and risk of premature release could limit their applications.

## Branched-tail ionizable lipids

Along with tail length and saturation, tail branching can greatly influence ionizable lipid performance. Ionizable lipids with methacrylate tails (a 1C branch near the head) generally showed reduced efficacy compared to acrylate-based ones^39^. Interestingly, ionizable lipids containing isodecyl acrylate (O_i10_, a 1C branch at the end) dramatically boosted hepatic mRNA expression (>10-fold) compared to their isomers with linear tails, despite their similar biodistribution^40^. This increased potency can be explained by enhanced endosomal escape due to the stronger protonation of spaced ionizable lipids at endosomal pH and the increased cross-section of the lipid tails, allowing for adoption of a more cone-shaped structure.^41^ The lead ionizable lipid (**306O**_**i10**_) efficiently co-delivered multiple RNA constructs to the liver and transfected >80% of hepatocytes, Kupffer and endothelial cells, demonstrating the potential for integrating different therapeutic modalities (e.g., gene silencing, expression, and editing) to treat liver dysfunction^42^.

Recently, Dong and coworkers developed ionizable lipids with branched ester chains that had higher transfection efficiency in the liver than their analogs with linear ester chains, presumably due to the slower degradation rate of the secondary ester^10,31^. Their top-performing ionizable lipid (**FTT5**) demonstrated efficient delivery of large mRNA constructs for protein supplementation and base editing therapies. Notably, increased tail branching is one of the major features investigated by LNP companies^31^.

## Clinical development of ionizable lipids

**Fig. 2:**
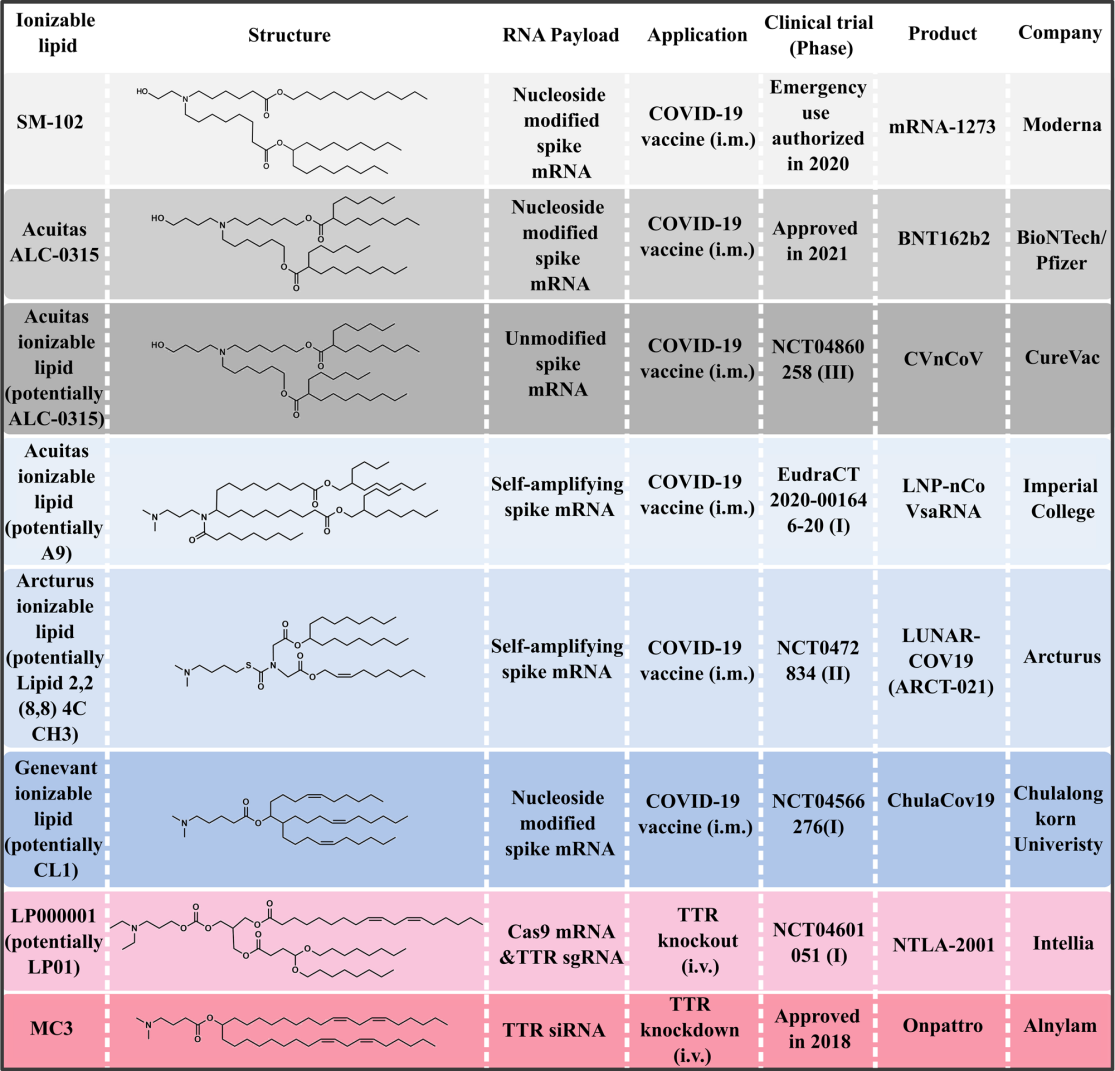
Selected ionizable lipids under clinical development for COVID-19 mRNA vaccines and other RNA therapeutics. Ionizable lipids used in on-going clinical trials have not been publicly disclosed, so one of the possible structures (**Acuitas A9**^46^, Arcturus **Lipid 2,2 (8,8) 4C CH3**^47^, **Genevant CL1**^48^ and **LP01**^49,50^) is shown, respectively. i.v. intravenous, i.m. intramuscular.

Before the approval of Onpattro^®^ in 2018, scientists have screened numerous ionizable lipids and LNP formulations  for more than a decade. The success of **MC3** has re-ignited the enthusiasm for RNA delivery and greatly accelerated the clinical development of other LNP-based RNA therapeutics, particularly mRNA vaccines (Fig. 2). Specifically, the COVID-19 pandemic has caused millions of deaths. However, in less than one year, two LNP-based mRNA vaccines (mRNA-1273 and BNT162b2) underwent unprecedented speed of development and received the historic approval for emergency use after demonstrating protection efficacies above 94%^43,44^. Interestingly, Moderna’s **SM-102** and BioNTech’s Acuitas **ALC-0315** have some shared features, including a tertiary amine, branched tails and ester linkers. Moreover, both of them bear extended aliphatic branches, making them appear like multi-tail structures. Currently, additional  COVID-19 mRNA vaccines delivered by LNPs are undergoing clinical development. Although these ionizable lipid structures have not been publicly disclosed, the probable ones are shown based on available patents and literature (Fig. 2).

Recently, positive results from the first human trial of LNP-enabled CRISPR-Cas9 in vivo gene editing were disclosed^45^. A single dose of **LP000001**-formulated LNPs carrying sgRNA targeting transthyretin (TTR) and Cas9 mRNA led to a 87% reduction of serum TTR in patients with hATTR, and only few or mild adverse events were observed. This study heralds an era for LNP-mediated in vivo gene editing.

When reviewing the approved **MC3**, **SM-102**, and **ALC-0315** structures as well as those patented by Acuitas et al.^46–48,50^, the ester-based biodegradable structure appears in every ionizable lipid; the next most commonly shared features are the multi/branched-tail and unsaturated structures. This suggests that biodegradability is a key feature for the clinical translation of ionizable lipids. Additionally, increased branching or tail number is a common characteristic for newly developed ionizable lipids. However, these multi/branched-tail ionizable lipids typically possess only one tertiary amine, which is a sharp contrast to those discussed in Fig. 1. Interestingly, while the unsaturated structure is important to ionizable lipids with long tails, it appears less important for those with multiple short tails.

## Challenges and future directions

Despite the FDA approval of ionizable lipids for RNA delivery applications, several challenges need to be addressed in order to reach the full potential of RNA therapeutics. First, the ionizable lipid is the key LNP component to cause acute immune responses and long-term toxicity. Although degradable ionizable lipids can be used to mitigate this issue, premedication with glucocorticoids and antihistamines before LNP infusion is still needed^45^. To overcome this issue, optimization of linker chemistry and inclusion of anti-inflammatory properties warrant further investigation^31,51^.

Second, the preparation of rationally designed ionizable lipid candidates is challenging due to the laborious synthesis process. For example, four synthetic steps and one week of intensive labor are required to synthesize **MC3**, and it is a huge burden to prepare all of its analogs^5^. Currently, clinical ionizable lipids are all synthesized by multiple steps, which results in scalable manufacturing challenges. This issue can be mitigated by adopting combinatorial chemistry for simplified  synthesis and accelerated ionizable lipid screening. However, top-performing ionizable lipids synthesized using this method usually contain stable backbones and multiple tertiary amines, leading to slow degradation and potential toxicity. Therefore, there is an urgent need to develop new combinatorial reactions that can generate degradable ionizable lipids for potent RNA delivery. Meanwhile, introducing additional functional modalities (e.g., STING activation) into these lipids would be of great value for specific RNA therapeutic and vaccine applications^9^.

Third, ionizable lipid-mediated extrahepatic delivery is another challenge to the broad application of RNA therapeutics. After systemic administration, neutral LNPs mainly localize in the liver regardless of the ionizable lipid^52^. While most ester-based ionizable lipids are reported to mainly transfect the liver, **OF-Deg-Lin** exhibits predominant splenic transfection^33^. Further disclosure of the relationships between structure and organ selectivity would greatly benefit extrahepatic RNA delivery. Besides passive targeting of extrahepatic tissue, rationally designed ionizable lipids with targeting ability can be promising for improving non-liver delivery. For example, a neurotransmitter-derived ionizable lipid has been shown to retain its active transporting ability to cross blood–brain barrier (BBB), allowing previously BBB-impermeable LNPs to successfully deliver ASOs into the brain^53^.

In summary, based on current research progress and clinical status, degradable backbones and increased branching/tails are two of the most favorable structural properties for the future development of ionizable lipids. Apart from safety and potency, next-generation ionizable lipids with additional functionalities such as targeting and immunomodulation will be important for specific applications. Therefore, there are still vast opportunities for ionizable lipid optimization and innovation to enable the broader translation of RNA therapeutics and vaccines.
